# Human brucellosis and fever of unknown origin

**DOI:** 10.1186/s12879-022-07872-8

**Published:** 2022-11-21

**Authors:** Zhi-guo Wu, Zhi-ying Song, Wei-xin Wang, Wen-na Xi, Di Jin, Mao-xing Ai, Yu-chan Wu, Yu Lan, Shu-fen Song, Gong-chang Zhang, Xue-bing Yao, Zhen Gao, Cui-yun Liu, Ke Sun, Dong-shan Yu, Bao-gang Xie, Shui-lin Sun

**Affiliations:** 1 Department of Infectious Diseases, The Second Affiliated Hospital of Nanchang University, Nanchang, 330006 China; 2 Department of Pharmaceutics, Medical College of Jiaxing University, Jiaxing, 314001 China; 3 Department of Infectious Diseases, The Second Affiliated Hospital of Yichun University, Yichun, 336000 China

**Keywords:** Brucellosis, Epidemiology, Misdiagnosis rate, Fever of unknown origin

## Abstract

**Background:**

Human brucellosis has become one of the major public health problems in China, and increases atypical manifestations, such as fever of unknown origin (FUO), and misdiagnosis rates has complicated the diagnosis of brucellosis. To date, no relevant study on the relationship between brucellosis and FUO has been conducted.

**Methods:**

We retrospectively reviewed the medical charts of 35 patients with confirmed human brucellosis and prospectively recorded their outcomes by telephone interview. The patients were admitted to the Second Affiliated Hospital of Nanchang University between January 01, 2013 and October 31, 2019. Patient data were collected from hospital medical records.

**Results:**

The percentage of males was significantly higher than that of female in FUO (78.95% vs. 21.05%, *P* < 0.05), and 80% of the patients had a clear history of exposure to cattle and sheep. Moreover, 19 (54%) cases were hospitalized with FUO, among which the patients with epidemiological histories were significantly more than those without (*P* < 0.05). The incidence of toxic hepatitis in FUO patients was higher than that in non-FUO patients (89% vs. 50%, *P* < 0.05). Meanwhile, the misdiagnosis rate was considerably higher in the FUO group than in the non-FUO group (100% vs. 63%; *P* < 0.05).

**Conclusion:**

Brucellosis is predominantly FUO admission in a non-endemic area of China, accompanied by irregular fever and toxic hepatitis. Careful examination of the epidemiological history and timely improvement of blood and bone marrow cultures can facilitate early diagnosis and prevent misdiagnosis.

## Background

Brucellosis is one of the most prevalent bacterial zoonoses worldwide and is mainly spread by sick domestic livestock (sheep, goats, cattle, camels, and pigs) and wild animals through consumption of their raw dairy products and infected meat and close contact with their secretions and carcasses [1–3]. Brucellosis accounts for more than 500,000 new cases per year globally and more than 10/100,000 morbidities in some endemic countries [4, 5]. According to the national surveillance data, the incidence of Brucellosis in China increased rapidly from 0.028/100,000 (326 cases) in 1993 to 3.1532/100,000 (44,036 cases) in 2019 [6, 7].

Brucella spp. survives for weeks or even months on environmental surfaces and enters the host through respiratory mucosa, conjunctiva, gastrointestinal tract, and worn skin, affects several organs and tissues, and leads to various clinical presentations that cause misdiagnosis [8–10]. Given the atypical symptoms of brucellosis, it is difficult for clinicians to distinguish a group of diseases with similar symptoms. Although in recent years Hamidi et al. [11] and Kazemi et al. [12] showed that the discovery of some potential markers can play an effective role in the rapid diagnosis of brucellosis. However, at present, these methods are rarely actually used in clinical practice, especially in non-endemic areas of brucellosis. Many patients with brucellosis present fever of unknown origin (FUO), which increases the difficulty of diagnosis. To the best of our knowledge, the relationship between brucellosis and FUO has not been explored. Thus, we focused on investigating this relationship to provide insights for the early diagnosis of the disease and prevention of its complications.

## Methods

### Setting

A total of 35 patients with laboratory-confirmed brucellosis were admitted to the Second Affiliated Hospital of Nanchang University from January 1, 2013 to October 31, 2019. All patients were included in this retrospective study. We accessed patient medical records and performed telephone interviews for more than 6 months. The data were examined using a standardized form, which was used in recording demographic data, medical history, clinical and laboratory findings, antibiotic treatment, and any focal involvements.

### Case definition

The confirmed cases of brucellosis were consistent with the diagnostic criteria [13]: The patients who met the following diagnostic criteria for brucellosis were included: (a) diagnosis was accompanied by clinical findings; (b) positive growth of *Brucella* species in the blood culture or any other body fluid or tissue cultures.

The exclusion criteria were as follows: (1) clinically diagnosed case; (2) patients who died due to inevitable factors during treatment; (3) Patients who did not complete treatment; (4) Patients who received unproven curative drugs or other drugs with unknown ingredients. FUO was defined as body temperature greater than 38.2 °C on three or more occasions and a duration of illness of at least 3 weeks without diagnosis despite 1 week of inpatient examination [14]. According to the clinical characteristics and treatment process, patients were divided into two groups: FUO group (n = 19) and non-FUO group (n = 16).

### Data analyses

Data were analyzed using IBM SPSS software version 25.0 (IBM Corp., Armonk, NY, USA). The distribution of variables in the groups was compared with the Chi-squared test or Fisher’s exact test for categorical variables. For normal distribution, independent sample t-test was used, and a value less than 0.05 was considered statistically significant.

## Results

### Demographic and epidemiological characteristics between FUO group (n = 19) and non-FUO group (n = 16)

#### Age profiles of patients with brucellosis

**Fig. 1:**
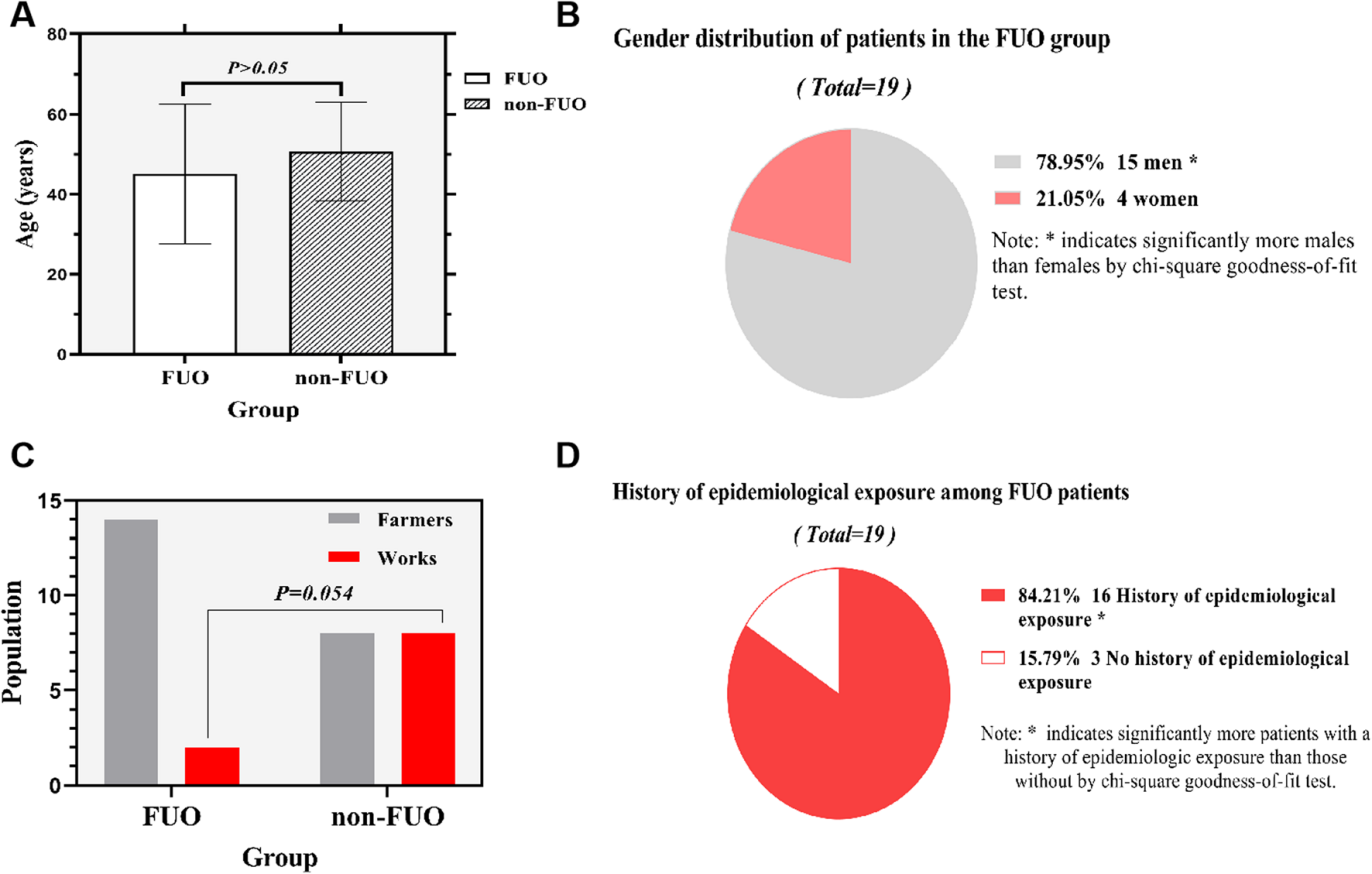
**A** Age profiles of patients with brucellosis, the mean age of the FUO group was 45.11 ± 17.49 years compared to 50.69 ± 12.37 years of the non-FUO group, and the difference was not significant (*P* > 0.05). **B** Differences in patient gender distribution in FUO group, the percentages of males were significantly higher than that of females (*P* < 0.05). **C** The percentages of farmers in the FUO and non-FUO groups were 73.68% (14/19) and 50.00% (8/16), respectively, and the percentages of workers were 10.53% (2/19) and 50.00% (8/16), respectively, showing no significant difference (*P* > 0.05). **D** Differences in patient epidemiological history in FUO group, patients with epidemiological history in FUO group were significantly more than those without epidemiological history (*P* < 0.05)

The 35 brucellosis patients were 17–71 years old with an average age of 47.66 ± 15.41 years; 19 patients were included in the FUO group, with an average age of 45.11 ± 17.49 years, and 16 patients were included in the non-FUO group, with an average age of 50.69 ± 12.37 years (Fig. 1A).

#### Significantly more male patients were in the FUO group

The patients composed of 27 (77.14%) males and 8 (22.86%) females, and the proportion between males and females was 3.38:1. The percentages of males and females in the FUO group were 78.95% (15/19) and 21.05% (4/19), respectively, and the number of male patients was significantly higher than that of female patients, showing significant difference (*P* = 0.012; Fig. 1B).

#### Farmers and workers are mainly affected by brucellosis

The prevalence rates in farmers, workers, cooks, butchers, and students were 62.86% (22/35), 28.57% (10/35), 2.86% (1/35), 2.86% (1/35), and 2.86% (1/35), respectively. The percentages of farmers in the FUO and non-FUO groups were 73.68% (14/19) and 50.00% (8/16), and the difference was not significant (*P* > 0.05). The percentages of workers in the FUO and non-FUO groups were 10.53% (2/19) and 50.00% (8/16), respectively, showing no significant difference (*P* = 0.054; Fig. 1C).

#### Significantly more patients with histories of epidemiologic exposure were in the FUO group

Approximately, 80% (28/35) of the patients had a history of epidemiological exposure to sheep and cattle, and 22 of them had a history of epidemiological exposure to sheep (including a case of drinking sheep blood, a case of touching sheep placenta, a case of touching sheep viscera, a case of eating dead mutton, and a case of eating uncooked mutton). Six had an epidemiological history with cattle (including a case of delivering cows). The percentages of epidemiological history with sheep and cattle were 62.86% (22/35) and 17.14% (6/35). The route of infection of another 20% (7/35) was unclear. The percentage of epidemiological history in FUO was 84.21% (16/19). The proportion of epidemiological history in FUO was significantly higher than that without epidemiological history, and the difference was significant (*P* = 0.003; Fig. 1D).

#### Onset of disease was mainly from April to June in both groups

The onset time of disease was mainly from April to June in 22 cases (62.86%), of which 12 (63.16%) were in the FUO group and 8 (50%) were in the non-FUO group. The difference between the groups were not significant (*P* > 0.05).

#### Fragmented distribution of patients’ residence

The residence distribution of the patients was scattered: 10 cases (28.57%) in Fuzhou, China. 6 cases (17.14%) in Yichun, China. 5 cases (14.29%) in Nanchang, China. 4 cases (11.43%) in Shangrao,China. 4 cases (11.43%) in Jiujiang, China. 3 cases (8.57%) in Yingtan, China. 2 cases (5.71%) in Xinyu, China. and 1 case (2.86%) in Ji’an, China.

### Clinical characteristics between FUO group (n = 19) and non-FUO group (n = 16)

The percentages of fever, fatigue, muscle arthralgia, shivering, and hyperhidrosis in FUO were 100%, 89.47%, 84.21%, 57.89%, and 52.63%, respectively. Differences in these clinical characteristics were not significant between the FUO and non-FUO groups (*P* > 0.05; Table 1). The rates of toxic hepatitis in the FUO and non-FUO groups were 89.47% and 50.00%, respectively, and the difference was significant (*P* = 0.022; Table 1).

**Table 1:** Clinical symptoms and laboratory examination in FUO and non-FUO groups

	FUO	non-FUO	P
Symptoms			
Fever	19 (100.00)	15 (93.75)	0.457
Fatigue	17 (89.47)	13 (81.25)	0.379
Arthralgia	16 (84.21)	8 (50.00)	0.065
Chills	11 (57.89)	6 (37.50)	0.315
Sweat	10 (52.63)	4 (25.00)	0.166
Complications			
Toxic hepatitis	17 (89.47)	8 (50.00)	0.022
Laboratory examination			
WBC	5.84 ± 2.36	6.09 ± 2.39	0.770
EO	0.15 ± 0.28	0.79 ± 1.30	0.074
HGB	117.53 ± 17.21	119.75 ± 14.69	0.687
CRP	34.82 ± 19.01	41.75 ± 31.66	0.430
ESR	37.63 ± 22.59	39.73 ± 15.84	0.792
Widal Test	5 (35.71)	1 (9.09)	0.180
Misdiagnosis rate (%)	19 (100.00)	10 (62.50)	0.005
Tuberculosis and typhoid fever	9 (47.37)	2 (20.00)	0.234

### Laboratory tests between FUO group (n = 19) and non-FUO group (n = 16)

#### Routine laboratory examination

Among the 35 patients, 25 patients underwent Widal Test, 6 (24.00%) were false positives. The false positive rates of the Widal test were 35.71% and 9.09% in the FUO and non-FUO groups, respectively, and the difference was not statistically significant (*P* = 0.180). In addition, the differences in routine laboratory tests such as WBC, EO%, HGB, CRP, and ESR between the FUO and non-FUO groups were not statistically significant (*P* > 0.05; Table 1).

#### Bacterial cultures

Brucella spp. belongs to a group of small gram-negative spheroidal bacilli, which need high nutritional culture media and grow slowly. Blood cultures were performed in 34 patients, of which 31 were positive. Bone marrow cell culture were performed in 17 patients, of which 15 were positive. Bone marrow cell culture and blood cultures were performed in 16 patients, of which 14 patients (87.50%) were bone marrow cell culture positive, 13 patients (81.25%) were blood culture positive, and 11 patients (68.75%) were positive to both tests. The average positive time of blood culture was 5.73 days, and that of bone marrow cell culture was 5.36 days. The FUO group had an average positive time of 5.5 days for blood culture and 5.17 days for bone marrow cell culture. The non-FUO group had an average positive time of 6 days for blood culture and 5.6 days for bone marrow cell culture. No statistically significant differences in the mean positive time for blood culture and bone marrow cell culture were found between the FUO and non-FUO groups (*P* > 0.05). Moreover, no statistically significant difference between the mean positive time of bone marrow cell culture and the mean positive time of blood culture was found in both groups (*P* > 0.05).

### Misdiagnosis rate difference between the FUO group (n = 19) and non-FUO group (n = 16)

**Fig. 2:**
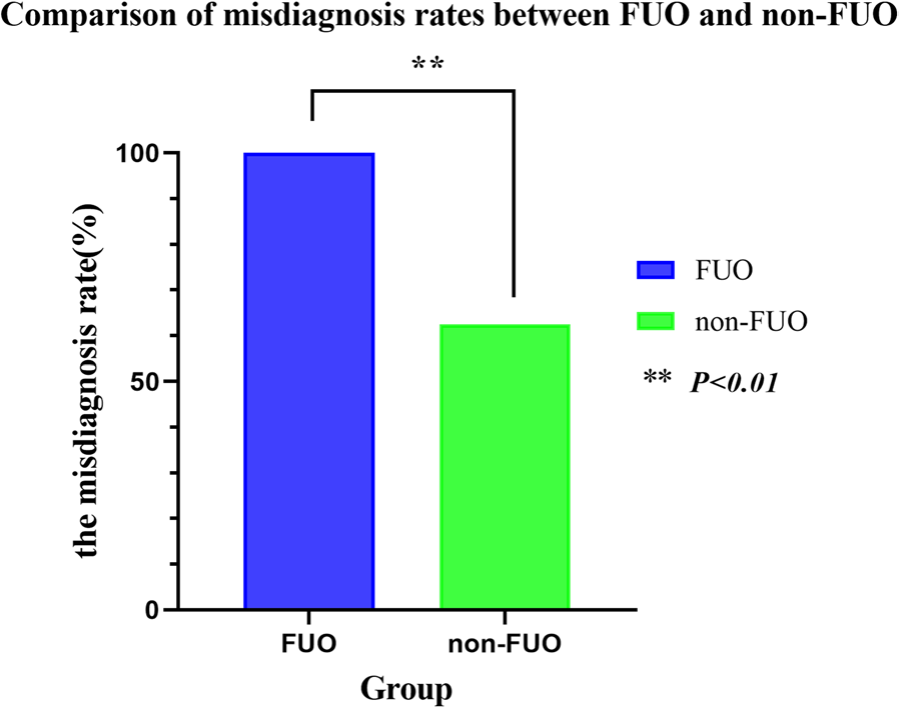
Misdiagnosis rate difference between the FUO and non-FUO groups was significant (100% vs. 62.50%, *P* < 0.01)

Among the 35 patients, 19 patients (54.29%) were hospitalized with the characteristic of FUO, and the misdiagnosis rate was 100% (19/19); 16 patients (45.71%) were hospitalized with the characteristic of non-FUO, and the misdiagnosis rate was 62.50% (10/16). The difference between the misdiagnosis rates of the FUO and non-FUO groups was significant (100% vs. 62.50%, *P* = 0.005; Fig. 2). The percentages of admitted patients with FUO misdiagnosed as common bacterial infection, tuberculosis, typhoid fever, viral infection, and non-infectious fever were 36.84%, 26.32%, 21.05%, 5.26%, and 10.53%, respectively. In the non-FUO group, the percentages of admitted patients misdiagnosed with bacterial infection, tuberculosis, typhoid fever, viral infection and non-infectious fever were 31.25%, 6.25%, 6.25%, 12.5% and 6.25%, respectively. Approximately 47.37% and 20.00% of the patients in the FUO and non-FUO groups, respectively, were misdiagnosed with tuberculosis and typhoid fever, and the difference was not statistically significant (*P* = 0.234; Table 1).

## Discussion

Brucellosis is a zoonosis caused by gram-negative Brucella spp. More than 500,000 new cases are reported annually globally, resulting in financial economic losses of up to 3 billion US dollars [15, 16]. In recent years, the epidemiology of brucellosis in China has changed dramatically, and the range of high incidence areas tends to move southward. Currently, the prevalence of brucellosis in old epidemic areas is still increasing, and new epidemic areas are gradually emerging [17, 18]. The trend is mainly due to the increased frequency of livestock breeding, inadequate surveillance and epidemic prevention, development in tourism, changing dietary habits, and lack of awareness of self-protection.

As an infectious disease, brucellosis can be transmitted to human beings and has a high degree of infectivity and morbidity. Owing to change in epidemic trend, sporadic epidemics have replaced large-scale outbreaks in recent years [19–21]. As a non-epidemic area, Jiangxi has no brucellosis outbreaks from 1976 to 2012 [22]. Since 2012, brucellosis cases have been reported continuously and showed an increasing trend every year. Brucellosis has atypical clinical symptoms and can thus mimic various multisystem diseases, such as those affecting the skeletal [23], nervous [24], blood [25], digestive [26], and cardiovascular systems [27]. Thus, it is often misdiagnosed and results in many complications. Fever is associated with many diseases, especially some infectious diseases, which have caused disasters, such as SARS and COVID-19, since the 21th century. Most infectious diseases are initially characterized by FUO, which remains one of the most difficult diagnostic challenges in medicine. Given that FUO may be caused by infectious, inflammatory, malignant, and miscellaneous disorders, clinicians often order non-clue-based imaging and specific testing early at the onset of FUO, and this approach may be inefficient or misleading [28]. In recent years, serious complications and even deaths from many diseases associated with FUO have been reported [29–31]. FUO is a persistent clinical problem that often confuses clinicians to the point that physicians need to deal with different diseases characterized by FUO at a great cost. Achievements in solving problems due to FUO have been minimal. We unexpectedly found that 54.29% (19/35) of brucellosis patients presented with FUO. Thus, for the purpose of early diagnosis and reduced complications of brucellosis, we focused on investigating the relationship between brucellosis and FUO.

In endemic countries, brucellosis is prevalent in the 15–35-year age group [10]. In our study, the age group was 18–65 years (57.14%), which constitutes the main labor force in China and has access to livestock. The mean age of patients in the FUO group was not higher than that in the non-FUO group. In patients with brucellosis, age may not be an independent risk factor influencing patients presented with FUO. Among the 19 patients of the FUO group, males were significantly higher in number than females (*P* = 0.012), indicating that FUO commonly occurred in male patients with brucellosis. In the present study, the affected patients were mainly farmers rather than butchers who had more access to livestock. The main reason was that butchers may have received safety training. Approximately, 80% (28/35) of the patients had a history of exposure to cattle and sheep. Notably, the number of patients with epidemiological history in the FUO group was higher than the number of patients without clear epidemiological history (*P* = 0.003). Epidemiological history plays an important role in the diagnosis of FUO, emphasizing the importance of determining history in clinical workup under any circumstance. Most patients (62.86%) had their onset in April–June, and the number of patients in the FUO group with onset during this time was higher than that in the non-FUO group. This period is close to the time of onset in endemic areas, as sheep mostly give birth in spring. Close contact with ewes may be the main reason for the higher number of patients with onset during this time [32, 33].

Brucellosis is a disease involving multiple systems and has diverse clinical manifestations [34]. In this study, fever (97.14%), fatigue (85.71%), muscle arthralgia (68.57%), chills (48.57%), and hyperhidrosis (40.00%) were the common symptoms. The percentage of patients with fever was 97.14%, higher than the percentages in previous studies [35–40]. The classic fever type of brucellosis is undulant fever [41]. However, only three of our patients (8.57%) presented with undulant fever, and the other 31 (88.57%) mainly presented with prolonged hypothermia and irregular fever. The reasons were antipyretic drugs and antibiotics used before admission according to the medical records and the telephone follow-up. The study of Jiang et al. [33] showed that most patients with FUO were admitted to hospitals, similar to our study. In addition, we found that the proportion of patients with toxic hepatitis was significantly higher in the FUO group than in the non-FUO group (*P* = 0.022), indicating that patients with FUO and brucellosis may be liver damage. We speculated that the main reason is that the FUO group had a longer duration of the disease and a significantly higher proportion of patients misdiagnosed with tuberculosis and typhoid than the non-FUO group. During treatment, antibiotics are commonly administered at local hospitals, and many of them are metabolized through the liver, causing liver damage.

In this study, the false positive rate of brucellosis Widal test was high (35.71% and 9.09% false positive rates in the FUO and non-FUO groups, respectively). This finding was not reported in previous studies. This result suggests that patients with brucellosis may develop a false-positive Widal test, and when brucellosis combined with FUO, patients may have a greater chance of developing a false-positive Widal test. However, large data collected over the next few years may provide stronger evidence for this assumption.

For brucellosis, the gold standard diagnostic assay is bacterial culture. In our study, the mean time to positivity of blood and bone marrow cell cultures was 5–6 days in both groups. Liu et al. [42] found that the positive rate of bone marrow culture was higher than that of blood culture and the lowest rate of cerebrospinal fluid positivity after analyzing brucellosis infection specimens in Peking Union Medical College Hospital from 2009 to 2018. In our study, the positive rate of bone marrow culture was 87.50% (14/16), and the positive rate of blood culture was 81.25% (13/16). No statistically significant difference was found (*P* > 0.05). Yang et al. [43] found that the positive alarm time for bone marrow culture was faster than that for blood culture, whereas Song et al. [44]. Concluded that the positive alarm times were similar and difference was not statistically significant. Our findings were similar to those of Song et al., and the difference in positive alarm time was not statistically significant (5.36 days vs. 5.73 days, *P* > 0.05). In addition, no statistically significant difference in the time to positive blood culture and bone marrow culture alarms was found between the FUO and non-FUO groups (5.5 days vs. 6 days; 5.17 days vs. 5.6 days; *P* > 0.05). No statistically significant difference between the time to positive bone marrow culture alarm time and positive blood culture alarm time was found in both groups (5.17 days vs. 5.5 days; 5.6 days vs. 6 days; *P* > 0.05). Given that the positivity of bacterial culture decreases with disease duration [45, 46], timely bone marrow cultures and blood cultures are important to the diagnosis of patients with brucellosis.

Brucellosis is a multisystem disease with a broad clinical spectrum and is one of the main reasons for the high rates of delayed diagnosis and misdiagnosis. In this study, 19 cases (54.29%) with FUO as a feature were admitted. We found that the rate of misdiagnosis was significantly higher in the FUO group than in the non-FUO group (100% vs. 62.50%; *P* = 0.005). The results of this study suggested that patients in the FUO group were more likely to be misdiagnosed and thus worthy of attention. Further study revealed that the percentages of patients misdiagnosed with tuberculosis and typhoid at initial diagnosis was 47.37% and 20.00% in the FUO and non-FUO groups, respectively. For the next study we may need larger data to confirm whether difference between the two groups is statistically significant.

## Conclusions

Human brucellosis is a multisystem disease with a broad clinical spectrum and is one of the main causes of delayed diagnosis and high misdiagnosis rates. The foundation of clinical diagnosis depends on detailed history and careful attention to epidemiological data. A detailed epidemiological history is particularly important to the diagnosis and exclusion of diseases with fever as a symptom. In addition, the majority of patients with FUO were admitted to hospitals. In areas where brucellosis is non-endemic, the possibility of the disease should be considered in patients with FUO and toxic hepatitis. Clinicians should fully understand the clinical characteristics of brucellosis and promptly perform blood and bone marrow cultures to help reduce misdiagnosis, missed diagnosis, and complications.

## Data Availability

All data during this study are included in this published article.

## Abbreviations

FUO: Fever of unknown origin
WBC: White blood cell
PLT: Platelet
HGB: Hemoglobin
EO: Eosinophil
CRP: C-reactive protein
ESR: Erythrocyte sedimentation rate
