# Correction: Human brucellosis and fever of unknown origin

**DOI:** 10.1186/s12879-023-07986-7

**Published:** 2023-01-12

**Authors:** Zhi-guo Wu, Zhi-ying Song, Wei-xin Wang, Wen-na Xi, Di Jin, Mao-xing Ai, Yu-chan Wu, Yu Lan, Shu-fen Song, Gong-chang Zhang, Xue-bing Yao, Zhen Gao, Cui-yun Liu, Ke Sun, Dong-shan Yu, Bao-gang Xie, Shui-lin Sun

**Affiliations:** 1 Department of Infectious Diseases, The Second Affiliated Hospital of Nanchang University, Nanchang, 330006 Jiangxi China; 2 Department of Pharmaceutics, Medical College of Jiaxing University, Jiaxing, 314001 China; 3 Department of Infectious Diseases, The Second Affiliated Hospital of Yichun University, Yichun, 336000 China

**Correction: BMC Infectious Diseases (2022) 22:868** 10.1186/s12879-022-07872-8

Following publication of the original article [1], the authors identified an error in Table 1. The correct Table 1 is given in this correction.

**Table 1:** Clinical symptoms and laboratory examination in FUO and non-FUO groups

	FUO	Non-FUO	P
Symptoms			
Fever	19 (100.00)	15 (93.75)	0.457
Fatigue	17 (89.47)	13 (81.25)	0.379
Arthralgia	16 (84.21)	8 (50.00)	0.065
Chills	11 (57.89)	6 (37.50)	0.315
Sweat	10 (52.63)	4 (25.00)	0.166
Complications			
Toxic hepatitis	17 (89.47)	8 (50.00)	0.022
Laboratory examination			
WBC	5.84±2.36	6.09±2.39	0.770
EO	0.15±0.28	0.79±1.30	0.074
HGB	117.53±17.21	119.75±14.69	0.687
CRP	34.82±19.04	41.75±31.66	0.430
ESR	37.63±22.59	39.73±15.84	0.792
False positive of Widar's	5 (35.71)	1 (9.09)	0.180
Misdiagnosis rate (%)	19 (100.00)	10 (62.50)	0.005
Misdiagnosed as tuberculosis and typhoid fever	9 (47.37)	2 (20.00)	0.234

The authors apologise for this error.

The original article [1] has been corrected.
